# Detection of exogenous siRNA inside sweet corn bundle sheath cells and the RNAi dynamics in the early stage of *Maize dwarf mosaic virus* infection

**DOI:** 10.1007/s12298-024-01500-2

**Published:** 2024-08-14

**Authors:** Kinga Balassa, György Balassa, Asztéria Almási, Tamás Visnovitz, Szabolcs Rudnóy

**Affiliations:** 1 Doctoral School of Biology, Institute of Biology, Eötvös Loránd University, Budapest, 1117 Hungary; 2 Department of Plant Physiology and Molecular Plant Biology, Faculty of Science, Eötvös Loránd University, Budapest, 1117 Hungary; 3 Department of Plant Pathology, Plant Protection Institute, HUN-REN Centre for Agricultural Research, Budapest, 1022 Hungary; 4 Institute of Genetics, Cell and Immunobiology, Semmelweis University, Budapest, 1085 Hungary; 5 Eurofins BIOMI Ltd, Gödöllő, 2100 Hungary

**Keywords:** ALEXA488, fluorescence microscopy, MDMV, Plant antiviral defence, SiRNA, Sweet corn

## Abstract

*Maize dwarf mosaic virus* (MDMV) is one of the most serious viruses of sweet corn. Utilising the process of RNA interference, the exogenous introduction of small RNA molecules mimicking virus-derived small interfering RNA (siRNA) into the plant prior to infection triggers the antiviral RNA silencing effect, thereby promoting more effective antiviral protection. Hence, a treatment with MDMV-derived small RNA was applied to sweet corn plants one day before MDMV virus inoculation. ALEXA FLUOR®488 fluorophore-bound exogenous siRNA was successfully detected inside intact sweet corn cells using confocal fluorescence microscopy. Furthermore, it was demonstrated that the exogenous siRNA treatment led to a notable upregulation of the *AGO1*, *AGO2b*, *AGO10b*, *AGO18a*, *DCL1*, *DCL3a*, *DCL4*, *RDR1*, and *MOP1* genes within 24 h of the treatment. Overall, exogenous siRNA treatment resulted in better virus control of infected sweet corn plants, as indicated by the lower viral RNA and coat protein levels compared to the infected group without pre-treatment.

**Supplementary Information:**

The online version contains supplementary material available at 10.1007/s12298-024-01500-2.

## Introduction

In cultivated crops significant yield losses are often caused by viral infections, such as *Maize dwarf mosaic virus* (MDMV), which is an important pest of sweet corn worldwide, causing up to 70% yield losses (Kannan et al. 2018). MDMV is classified into the *Potyvirus* genus of the *Potyviridae* family, which contains a total of 195 species, making it one of the most abundant plant-infecting virus genus (https://ictv.global/taxonomy). MDMV typically spreads mechanically, often vectored by aphids, and the main macroscopic symptoms that arise after systemic infection in plants include a mosaic-like arrangement of chlorotic spots and stunted growth, significantly reducing crop yield. If synergistic co-infection occurs, i.e. susceptible maize plants are simultaneously infected by MDMV and a virus from the *Machlomovirus* genus (*Tombusviridae* family), the deadly maize lethal necrosis (MLN) disease develops (Redinbaugh and Stewart 2018).

Plant RNA interference (RNAi) is pivotal for warding off virus infections, including *Potyvirus* and MDMV infections (Hameed et al. 2017; Gao et al. 2019). In this process, the assistance of the plant RNA-dependent RNA polymerase (RDR) and Dicer-like (DCL) proteins results in the accumulation of small interfering RNA (siRNA) molecules (Xia et al. 2014). These are then loaded into the AGO protein (AGO1, AGO2) located in the RISC complex, which promotes the binding and sequence-specific cleavage of the target viral mRNA (Hong et al. 2021). In this way, they contribute to reducing the amount of cytoplasmic viral mRNA, as well as to the prevention of further replication of the virus (Jin et al. 2021).

Taking advantage of the basic principle of the RNAi process, treatment with longer double-stranded RNA molecules activates the plant defence system without representing a real source of danger (Dalakouras et al. 2020). Thus, upon the appearance of a real infection, a stronger stress response can develop in the plants and, as a result, viral reproduction can be delayed (Kaldis et al. 2018; Rego-Machado et al. 2020). A similar effect can be achieved in the longer term, for example, by using carrier-bound dsRNA (Mitter et al. 2017). The foliar uptake of dsRNA molecules faces many obstacles, such as the cuticle, the cell wall and the presence of nucleases. Additionally, due to the negatively charged, water-soluble nature of dsRNA, it cannot pass through these layers by itself, but only with the use of additional techniques such as wounding, high-pressure spraying, or abaxial stomata flooding (Bennett et al. 2020). The high activity of RNases within the plant further reduces the chance of dsRNAs entering, which can be eliminated by using nuclease inhibitors or special dsRNA-binding and stability-increasing carrier substances (Demirer et al. 2019; Schwartz et al. 2019).

Simultaneously, the spraying technique was also found to be effective when employing siRNAs. Dalakouras et al. (2016) successfully achieved transgene silencing in GFP (green fluorescent protein) expressing *Nicotiana benthamiana* plants. It was found that the exogenous siRNAs, approx 20 nucleotides in length, were more likely to induce local silencing in plants if the treatment targeted the apical meristem rather than mature leaves. Dubrovina et al. (2020) demonstrated a decrease in *NPTII* (*neomycin phosphotransferase II*) transcript levels in transgenic *Arabidopsis thaliana* rosettes treated with an in vitro synthesized siRNA-water solution containing no added agents and applied directly to the leaf surface. Meanwhile, the ACMV (African cassava mosaic virus) infection in *Nicotiana benthamiana* plants treated with exogenous siRNA derived from ACMV can also be reduced, as demonstrated by Mohamed et al. (2022). In our study, siRNA molecules derived from MDMV were introduced into the open leaf sheaths of maize plants via direct foliar application. The exogenous siRNA-water solution encountered a young, developing tissue environment where cells have pliable walls and a thin cuticle, potentially facilitating the uptake of RNA molecules.

Conventionally, single-molecule imaging, such as fluorophore labelling and its detection under a fluorescence microscope, has been used to detect cell constituents, proteins, and even small RNAs in plant tissues. In the case of plants, however, the autofluorescence of lignin or chlorophyll makes imaging difficult, as there may be interference with the single-molecule fluorescent signals. In this case, the construction to be tested must be carefully selected and planned (Guo et al. 2021). CYP3 fluorophore and fluorescein (YFP) labelling have already been used to detect dsRNA uptake and transport in both fungal and plant cells (Koch et al. 2016; Qiao et al. 2021).

The aim of the current research was to investigate the effects of exogenous siRNA pretreatment on antiviral defence in the first week following the treatment in a previously established maize—MDMV model system. The main goal was to demonstrate whether the exogenous siRNA truly affected the expression of the genes involved in the RNAi process and whether this treatment could effectively reduce the viral load within the plant. For this, expression analysis was performed on 16 genes (3 *RDR*, 3 *DCL*, 10 *AGO*) related to the RNAi system of sweet corn, in addition to monitoring changes in the amounts of viral RNA and coat protein 1, 3 and 5 days after the first MDMV inoculation. A further objective was to ascertain whether the siRNA could be detected in intact cells using a microscopic method. Hence, the presence of siRNA in samples taken from the treatment sites was investigated with a fluorescent laser scanning microscope, in order to detect the specific signal of the ALEXA FLUOR® 488 fluorophore bound to MDMV CP-derived siRNA.

## Experimental procedures

### Plant material, growth conditions and treatments

Sweet corn (*Zea mays* cv. *saccharata* var. Honey Koern.) plants were used to examine the effect of siRNA treatment in the early stage of MDMV infection. After 3-day germination, corn grains were grown hydroponically on 1⁄4 strength Hoagland solution (containing 80 μM Fe(III)-EDTA as the iron form). Plant growth took place at 250 μmol photon m^−2^ s^−1^ PPFD, 23 ± 1 °C temperature and 50% relative humidity, in a SANYO MLR-350 HT (SANYO Electric Co., Ltd., Japan) plant growth chamber with a 14/10 h light/dark period. Plants without subsequent treatments were indicated as control (Co) plants. To investigate the effect of the siRNA (pre)treatment on RNA interference, 10-day-old plants were treated with 21-nucleotide siRNA molecules (IDT, Coralville, IA, USA) in the *siRNA* group. 10 μl of 30 ng/μl siRNA solved in MQ was pipetted into the open leaf sheaths. We aimed to create conditions similar to spraying using this non-invasive treatment method. During the foliar uptake, the droplet had dried on each plant by the following morning, making it a 24-h treatment before infection. The sequence of the siRNA (appendix Table 2) was identical to a 21-nt sequence in the 5’ part of the coat protein gene of reference MDMV genomes (AM490848, AM490849, FM883181, FM883202). This sequence was determined by means of small RNA sequencing on MDMV-infected sweet corn (unpublished). In addition, as a negative control, plants treated with sterile, nuclease- and siRNA-free MQ were examined at the same time and location. The first and second leaves of sweet corn plants from the infected*, *Inf group were inoculated mechanically with the MDMV Dallas A strain on two occasions, 11 and 13 days after germination. For this purpose, 1 g leaf tissue with macroscopic symptoms, taken from previously infected plants, was homogenized in 10 mL Sörensen phosphate buffer (pH 7.2, 0.06 M) and carborundum. This homogenate was used to inoculate healthy plants. To investigate the effects of exogenous MDMV CP-derived siRNA treatment in infected plants, the siRNA-pretreated plants were infected with the same MDMV strain (henceforth referred to as the *siRNA-Inf* group). The experimental setup and sampling times are shown in Supplementary Fig. 1.

### Detection of fluorescently labelled siRNA by confocal fluorescence microscopy

siRNA molecules conjugated with fluorophore (IDT, Coralville, IA, USA) were used to prove the entry of siRNA into the plant cells. To obtain the best signal-to-noise ratio, the autofluorescence of the targeted corn tissues was analysed (Supplementary Fig. 3 and Supplementary Fig. 4) and the ALEXA FLUOR® 488 (NHS Ester) fluorophore (IDT, Coralville, IA, USA, absorbance max: 492 nm, emission max: 517 nm), which was bound to the 3’ end of the siRNA sense strand, was selected for the study. The sequence of the fluoro-siRNA (appendix Table 2) matched the unlabelled siRNA used in the experiments, and the treatment protocol was also the same. Multiple transversal sections were obtained at a distance of 3, 5 and 10 mm from the open leaf sheath of plants treated with fluoro-siRNA. Sampling for the microscopic analysis was conducted concurrently with the first sampling for the qPCR examination, preceding the MDMV inoculation of the 11-day old plants. The tissues were mounted in 70% glycerol and were examined with a Leica SP8 lightning-confocal microscope. The ALEXA488 fluorophore was excited at a wavelength of 488 nm and its fluorescence was detected in the 545–555 nm range. Chlorophyll molecules were excited at 638 nm and detected in the 645–720 nm range. A 40 × water immersion objective (NA: 1.1) was used for the measurements, and LAS X (Leica) software for image analysis.

### Quantification of virus particles in the leaves of infected plants

To accurately monitor the development of MDMV infection, virus accumulation was examined in two ways: changes in the amounts of both viral coat protein and viral genomic RNA were monitored. MDMV coat protein was detected in sweet corn leaves with DAS ELISA (Clark and Adams 1977) using an MDMV antiserum kit (Bioreba A.G., Reinach, Switzerland) following the manufacturer’s instructions. Samples were taken from the third leaves of 11-, 12-, 14- and 16-day-old sweet corn seedlings, i.e. 0, 1, 3 and 5 days after the first inoculation (dpi—days post infection). Sampling from 11 days old plants preceded the MDMV inoculation. The amount of viral coat protein in each sample can be deduced from the different absorbance values determined at a wavelength of 405 nm with a Labsystem Multiskan MS spectrophotometer. The quantitation of viral RNA was performed using absolute quantification real-time PCR with the use of an MDMV-specific PrimeTime probe (IDT Integrated DNA Technologies, Coralville, IA, USA), a GoTaq® Probe qPCR Master Mix (Promega, Madison, WI, USA) and specific primers (appendix Table 3) designed for the MDMV genome 6K2 protein coding sequence (NCBI reference number: NC_003377.1). The qPCR reactions were run on an ABI StepOnePlus Real-Time PCR instrument (Thermo Fisher Scientific, Rockford, IL, USA) and the qPCR program setup was: 95 °C 2 min, and 40 cycles at 95 °C for 15 s, 60 °C for 1 min. The absolute determination was made using a calibration line, for which a synthesized 500 bp reference segment from the MDMV genome (CAA04929.1) was used. A six-member dilution series was prepared with this reference section and the results were used to compile a calibration curve (Supplementary Fig. 5). The equation (f(x) = − 1.729 ln(x) + 17.682; R^2^ = 0.995) of this calibration line was used to assign a concentration to each C_T_ value recorded for the plant samples. The results were expressed as viral RNA concentration (attomol/μl).

### Analysis of gene expression in maize

Total RNA was isolated from the third leaves of 11-, 12-, 14- and 16-day-old sweet corn seedlings, i.e. 0, 1, 3 and 5 days after the first inoculation (dpi—days post infection) using a Direct-zol RNA Miniprep Kit (Thermo Fisher Scientific), including the DNA digestion step. Sampling from 11 days old plants preceded the MDMV inoculation. The purity and concentration of the RNA samples were checked using a Jenway Genova Nano spectrophotometer (Bibby Scientific Ltd., Stone, Staffordshire, UK). cDNA was synthesised from 500 ng RNA with a RevertAid First Strand cDNA Synthesis Kit (Thermo Fisher Scientific) with the use of random hexamer primers. The qPCR reactions were run on an ABI StepOnePlus Real-Time PCR instrument (Thermo Fisher Scientific), using a Maxima SYBR Green/ROX qPCR Master Mix (Thermo Fisher Scientific). Three housekeeping genes, folylpolyglutamate synthase (*FPGS*), leunig (*LUG*) and membrane protein PB1A10.07c gene (*MEP*) were used as internal control genes to normalize the Cq values of the studied genes. The relative gene expression changes were first compared with the untreated control group and then quantified using the Pfaffl method (Pfaffl 2004). The primers used for the qPCR reactions were designed on Primer 3 online software (https://primer3.ut.ee/; Koressaar and Remm 2007). The properties of the reference and examined gene primers (name, sequence, amplicon length and reaction efficiency) are listed in appendix Table 1. The reaction efficiency of the primers was determined using LinReg software (Ramakers et al. 2003). The gene expression analysis also included the most important RNAi protein genes used in virus control: the RNA-dependent RNA polymerase (RDR), the dicer-like (*DCL*) and the argonaute (*AGO*) genes. The average log_2_ relative gene expression values for these genes, based on three biological and three technical replicates, are available in Table SI4.

### Data evaluation and statistical analysis

Three technical repeats and three biological repeats were used both for the qPCR experiments and for the quantification of virus particles. After checking the normality of the data the results were statistically evaluated with ANOVA and Tukey’s honest significant difference (TukeyHSD) post-hoc test at the 5% significance level (*p* ≤ 0.05) using RStudio (Racine 2012). The heatmap of the centred and scaled log_2_ relative expression values was generated using the pheatmap package in Rstudio (https://CRAN.R-project.org/package=pheatmap). The gene clusters were classified by Z-score determination of each row. The expression data of the genes were compared using Pearson Correlation Coefficient analysis, which indicates negative and positive correlations between genes on a scale between − 1 and + 1. Significant differences were determined by classifying these data according to their *p*-values (*p* < 0.05 is indicated by one star and *p* < 0.01 by two stars). Fluorescence tests were optimized with two biological and two technical repetitions, while the measurements were performed with three biological and three technical repetitions.

## Results

### Detection of fluorophore-labelled siRNA in sweet corn bundle sheath cells

Based on the autofluorescence of targeted epidermal and parenchyma tissues (Supplementary Figs. 3 and 4) and the fluorophores available for siRNAs, the ALEXA488 fluorophore was selected for the examination. The wavelength range of detection (545–555 nm) was carefully chosen to avoid the cell autofluorescence from the epidermis and parenchyma and the signal to noise ratio in close vicinity of the emission (Supplementary Fig. 2).

**Fig. 1:**
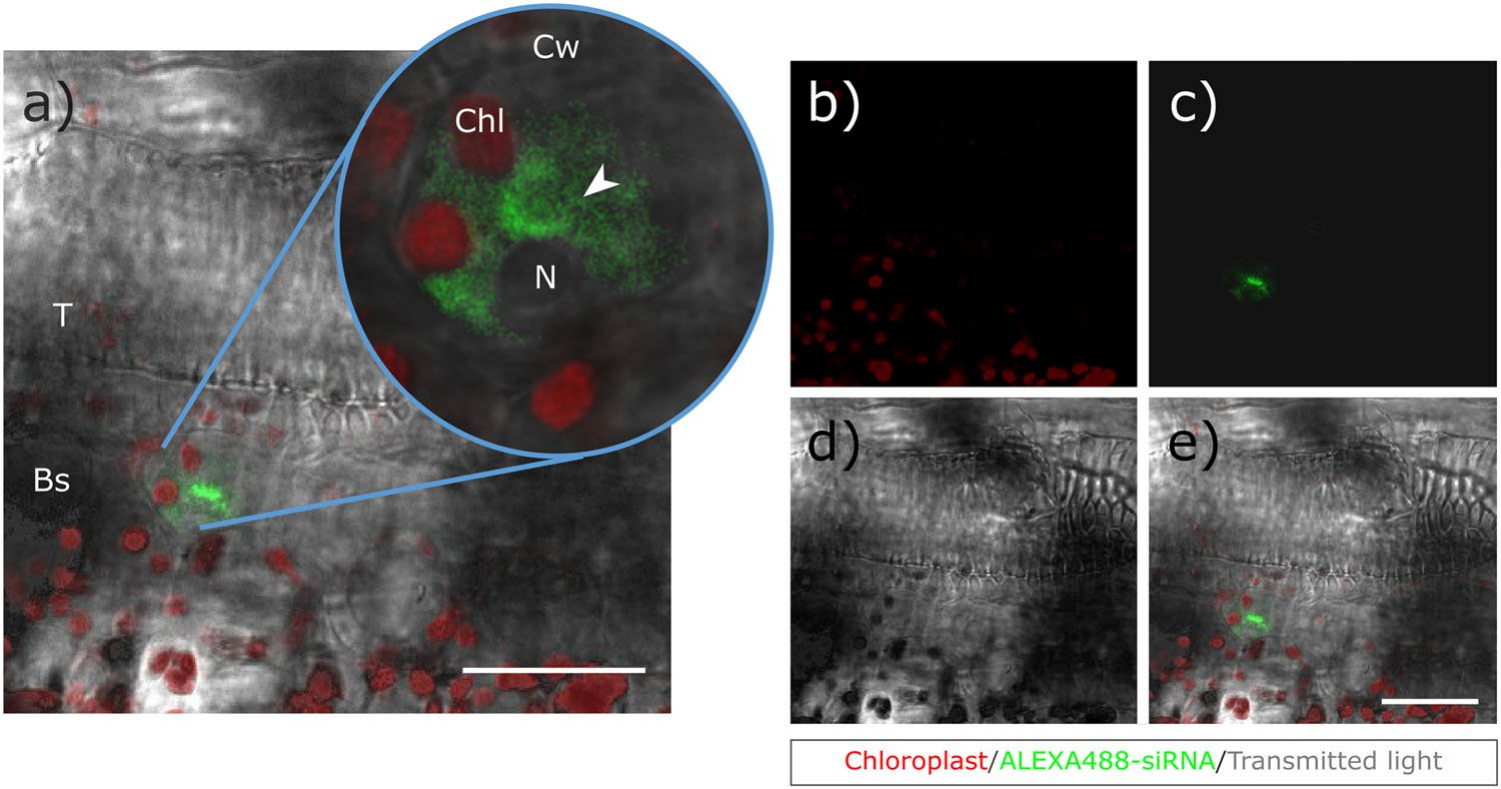
Confocal micrographs of sweet corn leaves treated with ALEXA488-linked siRNA. Green fluorescence shows the presence of siRNA. Red fluorescence indicates the chloroplasts. The fluorescence signals were merged with the transmitted light image to enable recognition of the cellular structure of the tissues. **a** Intracellular localisation of the siRNA. Specific green fluorescence can be clearly detected inside a bundle sheath parenchyma cell. Panels *b-e* represent the channels of the image at lower magnification. Background green fluorescence was not detected **c**. Scale bar represents 25 μm. (arrow—ALEXA488 signal, Bs—bundle sheath parenchyma, Chl—chloroplasts, Cw—cell wall, N—nucleus, T—trachea)

**Fig. 2:**
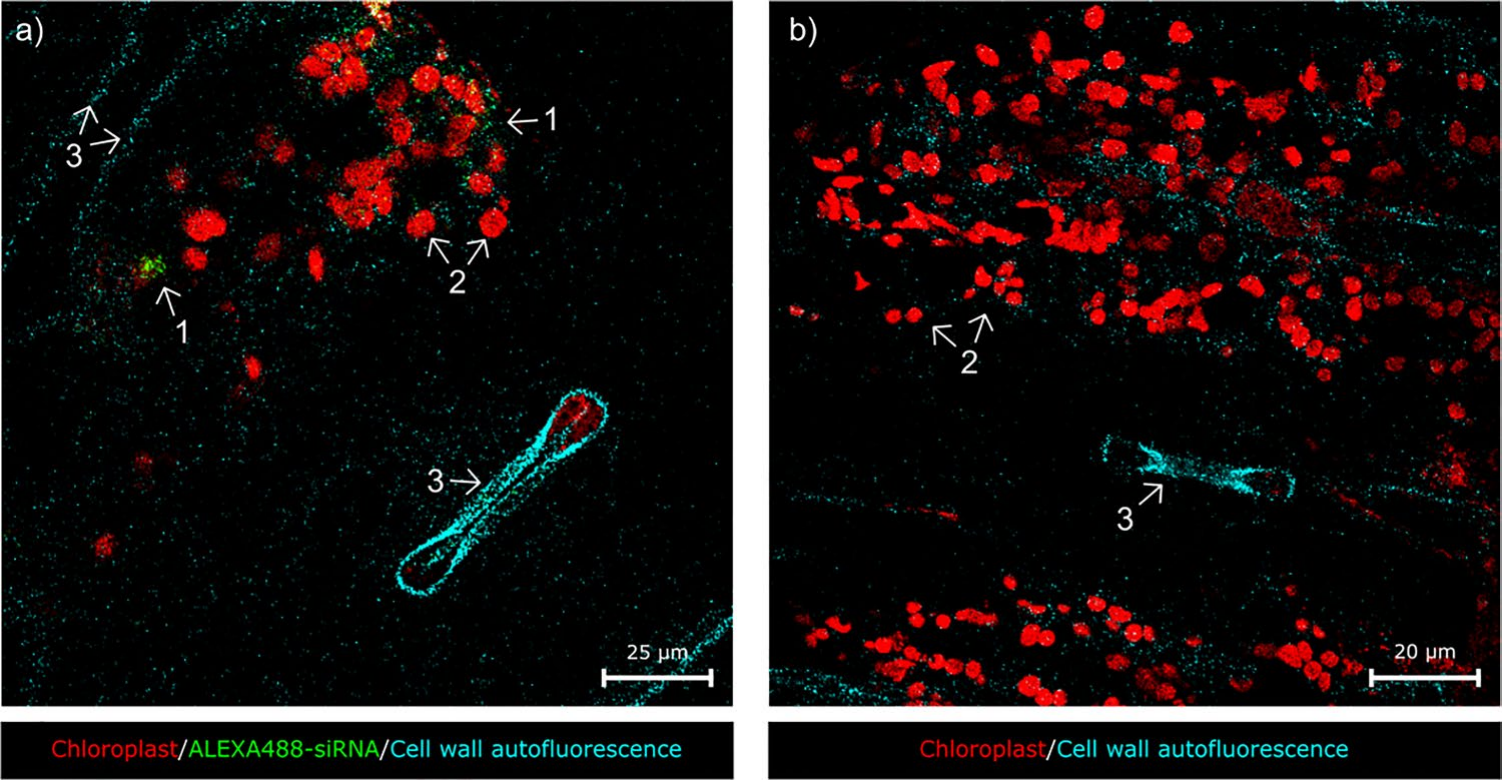
Confocal micrographs of **a** sweet corn leaves treated with ALEXA488-linked siRNA and **b** negative control samples that consistently lacked the ALEXA FLUOR 488® fluorescent signal. The numbers represent the different channels: 1—green, ALEXA488-siRNA fluorescence, 2—red, chloroplast autofluorescence, 3—cyan, cell wall autofluorescence, (red excitation 638 nm, red detection 645–720 nm; green excitation 488 nm, green detection 535–565 nm)

Transverse sections were made from each treated plant at 3, 5 and 10 mm below the small RNA treatment site. Among these sections, the ones closest to the treatment site (collected at a distance of 3 mm) showed a positive ALEXA488 signal, as shown in the animation (Online Resource 1) 20 h after the treatment. A total of 6 fields per section were analysed, about the half of which showed a positive signal in the 3 mm section. The specific ALEXA488 signal could be seen in the bundle sheath parenchyma cells of the transport tissue system of the treated plants, but no fluorescent signal was found inside the trachea or the phloem. Figure 1 shows a representative image of the accumulation of the specific ALEXA488 signal close to chloroplasts in an intact plant cell surrounded by a cell wall, all fields giving a positive signal were seen to be similar. 20 h after the treatment a fluorescent signal was only found in a few cells (Fig. 2a, Online Resource 2), which means that the recordings were made before systematic spread. In contrast to this, in a negative control, where plants were treated with sterile, nuclease- and siRNA-free MQ and were also examined at the same time and location, samples consistently showed a lack of ALEXA 488 fluorescence signals around the chloroplasts inside intact cells (Fig. 2b, Online Resource 3).

### Expression profiles of the sweet corn RNAi genes

**Fig. 3:**
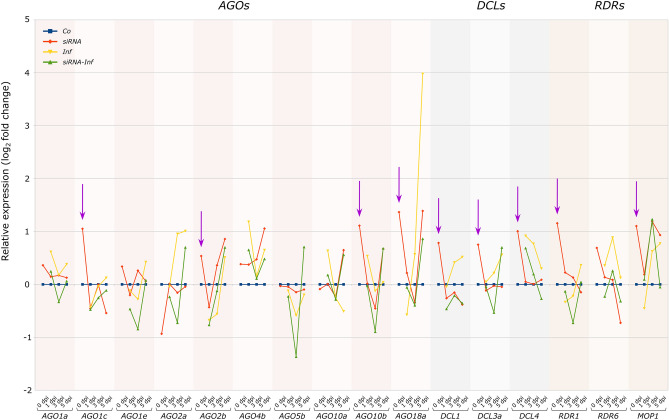
Relative gene expression changes in RNAi genes plotted on a log_2_ scale, value of the control group is 1 (log_2_1 = 0). Purple arrows indicate significant differences (p ≤ 0.05; Tukey’s HSD) for individual genes between the siRNA and the control (Co) group at 0 dpi. The mean values of the three biological and three technical replicates included on the graph can be found in the supplementary Supplementary Table 4 (*Co* – Control; *siRNA* – siRNA-treated group, *Inf* – MDMV-infected group, *siRNA-Inf* – siRNA-pretreated and MDMV-infected group)

Parallel to siRNA entry and MDMV infection, the key genes of antiviral RNA interference are activated in sweet corn seedlings. Throughout the examinations, alterations in expression were assessed for 16 genes associated with the RNAi mechanism (*AGO1a*, *AGO1c*, *AGO1e*, *AGO2a*, *AGO2b*, *AGO4b*, *AGO5b*, *AGO10a*, *AGO10b*, *AGO18a*, *DCL1*, *DCL3a*, *DCL4*, *MOP1*, *RDR1*, *RDR6*) across the treatment groups. These changes were depicted on a log_2_ scale graph (Fig. 3).

Based on the results, it was demonstrated that siRNA (pre)treatment influenced the gene expression of antiviral RNAi genes during the first week after treatment. This was shown by the significantly higher value of the *siRNA* group compared to the Control at 0 dpi, manifested in the expression intensity of all the *DCL* genes, as well as in most *RDR* and *AGO* genes. The most conspicuous trend observed as a result of the infection was the gradual increase in expression activity in a total of 8 genes (*AGO1c*, *AGO2a*, *AGO2b*, *AGO18a*, *DCL1*, *DCL3a*, *MOP1* and *RDR1*) in the *Inf* group. The second most characteristic trend emerging as a result of infection was the “V”-shaped expression change, which could only be observed among the *AGO* genes, affecting a total of 5 genes. This dual high early-late expression pattern, featuring a dip at the second sampling point, was observed for *AGO1a*, *AGO1e*, *AGO4b*, *AGO5b*, and *AGO10b*. In the case of the *AGO10a* and *DCL4* genes, a gradually decreasing trend was observed. In contrast, RDR6 exhibited an “A”-shaped trend, which indicates a generally low early-late gene expression interrupted by a moderately high activity at the second sampling point.

In addition to the outstanding values of the *siRNA* group at 0 dpi, the effect of siRNA treatment was also shown by the gene expression changes in the *siRNA-Inf* group at 1, 3 and 5 dpi. For 13 of the 16 genes examined, the expression value of the *siRNA-Inf* group was below that of the *Inf* group. In the case of the *AGO1a*, *AGO1c*, *AGO2a*, *AGO4b*, *AGO5b*, *AGO10b*, *AGO18a*, *DCL1*, *DCL3a*, *DCL4*, *RDR1* and *RDR6* genes, significant differences could also be observed on individual sampling days.

Among all the genes, the most remarkable expression change in *Inf* was recorded for *AGO18a*, which increased approximately four times compared to the *control*. The *siRNA-Inf* group on the other hand, where infection followed the siRNA pretreatment, closely followed the trend of the *siRNA* group, showing significantly lower expression compared to the *Inf* group.

**Fig. 4:**
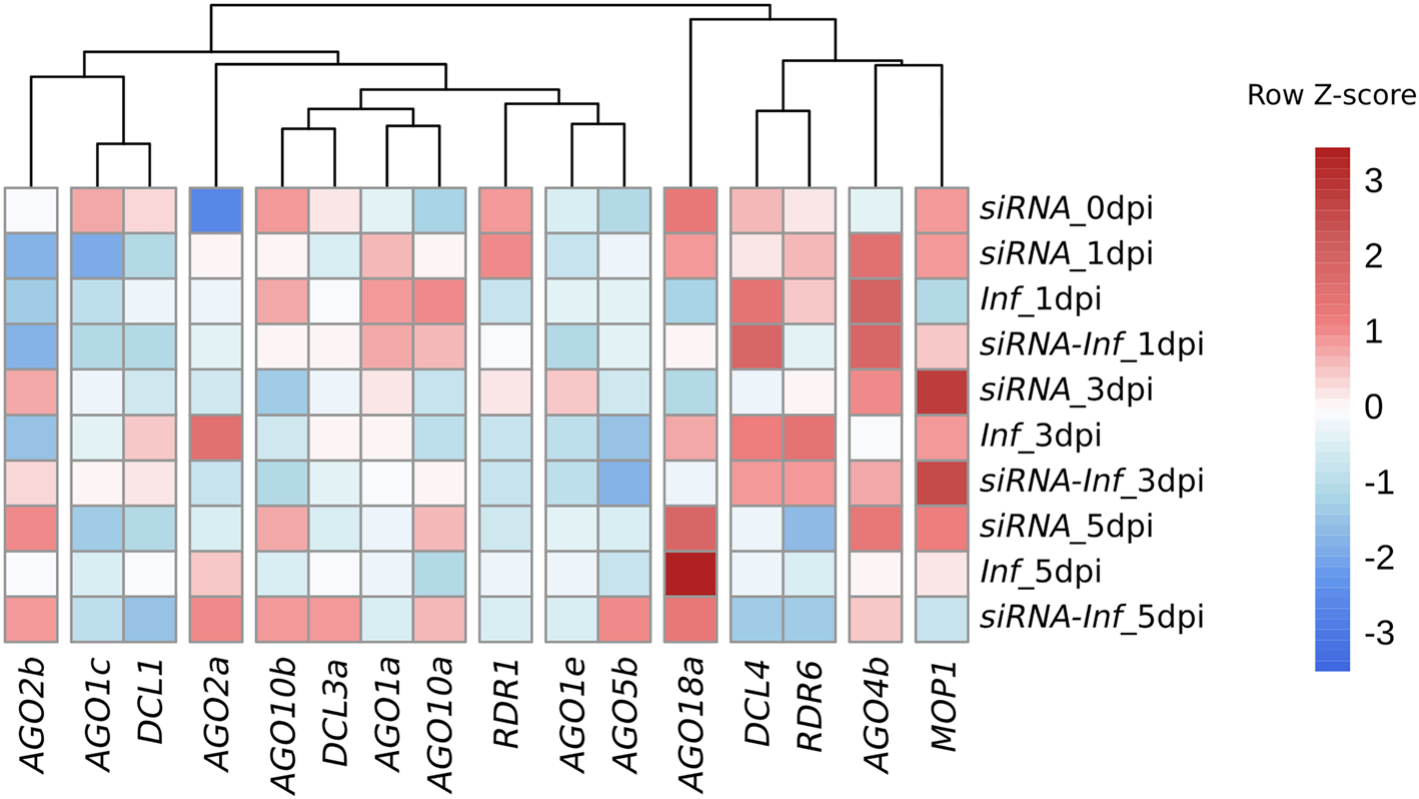
Heatmap of gene expression based on log_2_ relative expression data during the initial phase of MDMV infection with or without siRNA (pre)treatment. The data is based on the comparison of all treatment groups and time points, columns represent genes, while rows represent treatment groups at given time points. Genes were classified by determining their relative Z-scores in the rows. Red boxes indicate upregulation and blue boxes downregulation

Genes showing a similar expression trend over the time course of the different treatments can be classified into the same clusters on the gene expression heatmap (Fig. 4). Clusters with early activation, late inhibition, and continuous expression can be distinguished. For example, in the case of the *DCL4* and *RDR6* genes, after strong initial activity, a significant decrease occurred on the last measurement day. The *AGO1c*, *DCL1* and *RDR1* genes showed an early expression peak, which was later followed by reduced activity, while the *AGO4b* and *MOP1* genes exhibited continuously elevated expression. The most populous group included genes (*AGO1a*, *AGO10a*, *AGO10b*, *DCL3a*) that showed higher expression at the beginning and end of the studied timescale. According to the heatmap, the activity of *AGO18a* was clearly the most prominent.

**Fig. 5:**
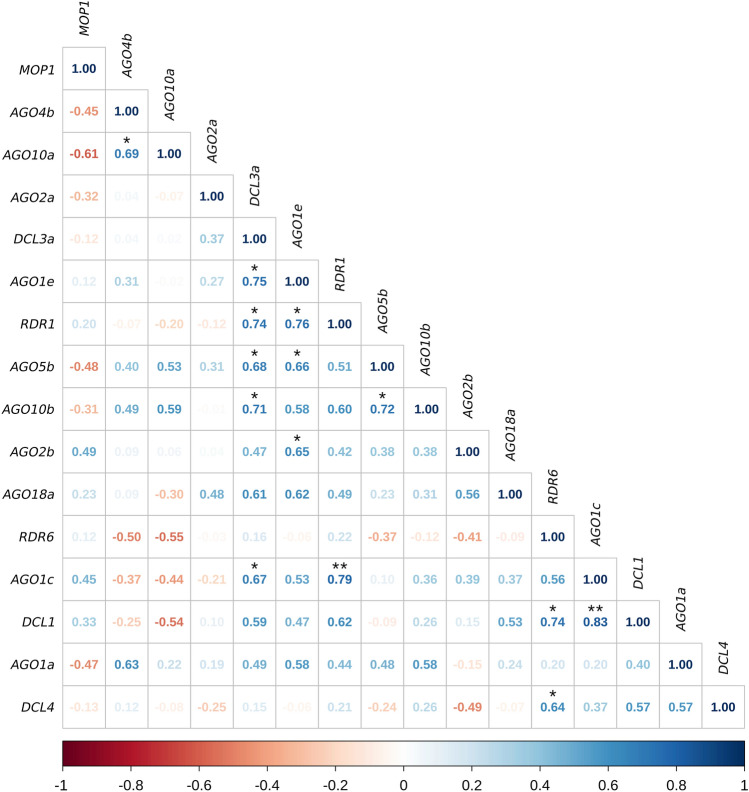
Pearson correlation matrix of the studied gene expression data based on the comparison of all treatment groups and time points. The data (r) were plotted on a scale of − 1 to + 1. Colours indicate positive and negative correlations, while numbers indicate the strength of the correlation between two genes (blue—positive correlation, white—no correlation, red—negative correlation; r < 0.25—no relationship, 0.25 < r < 0.5—weak relationship, 0.5 < r < 0.75—moderate relationship, r > 0.75—strong relationship). *−*p* < 0.05, **−*p* < 0.01

Pearson’s correlation analysis provides information about relationships between the expression of the genes (Fig. 5). The data in the correlation matrix obtained from the analysis of all sampling times overwhelmingly pointed in the direction of a positive interaction. For some gene pairs, there was no detectable relationship or a slight negative interaction, but no strong negative relationships were found, since r did not exceed -0.75. It should be noted that significant values were only found for genes showing a positive correlation. At a 95% significance level, a total of 14 pairings (p < 0.05) proved to be significant, of which the p-value of 2 pairs did not exceed 0.01. About half of the gene pairings showing a positive correlation were classified as weak and the other half as moderate relationships, while in the case of 3 pairings (*AGO1e*—*RDR1*, *AGO1c*—*RDR1*, and *AGO1c*—*DCL1*) a strong positive relationship was revealed. It was interesting to note that despite the central role of *AGO18a*, it only exhibited a weak or moderate interaction with the other genes.

### Quantification of virus particles in the leaves of infected plants

**Fig. 6:**
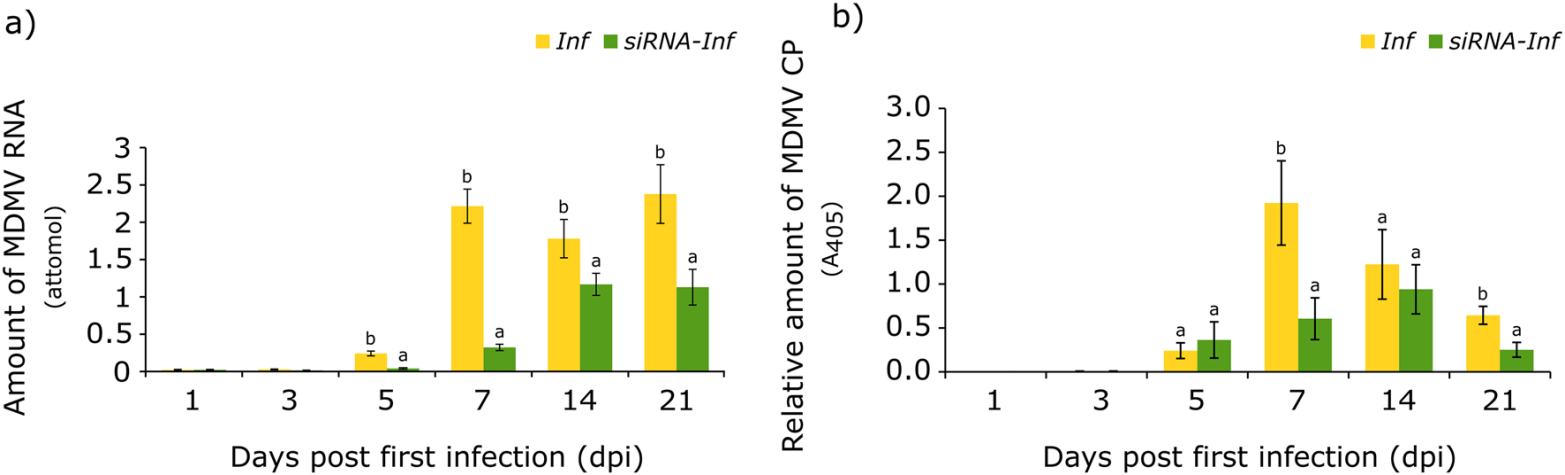
Determination of virus particle quantities during the experiment: **a** changes in MDMV RNA amounts measured with absolute quantification real-time PCR and **b** changes in MDMV coat protein amounts measured with DAS ELISA (*Inf—*MDMV-infected group, *siRNA-Inf* – siRNA-pretreated and MDMV-infected group). Error bars represent SD and the letters indicate statistically significant differences (*p* ≤ 0.05; Tukey’s HSD)

The first macroscopic symptoms of MDMV infection are yellow mosaic spots, which typically appear 3–4 days after the first inoculation. As the plant grows, the spotting continues to spread to the youngest new leaves, indicating a systemic infection in the plants. This could be seen in the increasing levels of RNA and coat protein observed at 7 dpi (Fig. 6). In order to get a more accurate picture of the initial stage of infection, changes in the amount of viral genetic material and coat protein in all the treatment groups were monitored during the first week after siRNA treatment. Neither MDMV RNA, nor coat protein were detectable in the non-infected groups (*Co*, *siRNA*). It can be seen that three days after the second inoculation, at 5 dpi, virus particles could be detected for the first time from the developing leaves of infected plants. On this day, the amount of MDMV RNA in the *Inf* group already significantly exceeded the value of the *siRNA-Inf* group (Fig. 6a). This difference persisted in the later stages of infection (7, 14, 21 dpi). At 5 dpi the amount of coat protein was practically the same in both groups, but the average value of the *siRNA-Inf* group remained below the value of the *Inf* group at all subsequent measurement points. It is important to note that, as a result of siRNA pre-treatment, the maximum value of coat protein in the *siRNA-Inf* group was significantly lower and peaked a week later compared to the Inf group (Fig. 6b).

## Discussion

The aim of the current study was to demonstrate the impact of exogenously derived siRNA on modulating the expression of RNAi genes in the early stages of MDMV infection, to establish the reduction in viral load as a consequence of the treatment, and to provide visible evidence of the presence of siRNA in sweet corn cells. The location of siRNAs after the treatment became detectable using the ALEXA488 fluorophore synthesised at the 3’ end of the sense strand of the MDMV-specific siRNA commonly used by the research team. Using confocal microscopy, the specific ALEXA488 signal was clearly visible in samples taken from siRNA-treated plants, while nothing similar was found in the negative control plants. In many cases the ALEXA488 signal was visible on the surface of the adaxial epidermis 20 h after treatment and also appeared in the mesophyll and/or bundle sheath parenchyma cells. Koukiekolo et al. (2009) described a FRET-based method capable of specifically detecting siRNA-protein interaction. The Cyan Fluorescent Protein (CFP) fluorophore-linked *Carnation Italian ringspot virus* p19 protein was capable of binding Cy3-labelled siRNA specifically, resulting in a significant decrease in CFP fluorescence. As a result of the fluorescence reduction, it became possible to quickly determine the amount of fluorophore-linked siRNAs in solution. Improvements in the technology now make it possible to detect fluorophore-labelled siRNAs in intact plant cells. For example, Demirer et al. (2020) used fluorophore-bound, Green Fluorescent Protein (GFP)-specific siRNA to demonstrate the efficacy of their newest single-walled carbon nanotube (SWNT)-based siRNA delivery platform in transgenic *mGFP5 Nicotiana benthamiana*. The success of the SWNT platform was indicated by the fact that the Cy3-siRNA signal was detectable from intact cells as little as 6 h after treatment. Moreover, significant GFP-Cy3 signal colocalization was observed, causing the suppression of the GFP signal in plants treated with siRNA-SWNT. They also found that nanotube-bound single-stranded siRNA was protected from degradation, as it was still detectable in intact cells after 24 h. In addition, the presence of miRNA was proved by fluorescence microscopy. For example, Betti et al. (2021) demonstrated the presence of exogenous, Cy3 fluorophore-bound *miR399* from intact xylem cells in *Arabidopsis thaliana*.

Small RNAs are able to spread within the plant, thus contributing to the plant’s systemic antiviral defence (Brosnan and Voinnet 2011). siRNA molecules use the same pathway along which the virus moves within the plant during infection (Hipper et al. 2013). Inside the cells, in a symplastic way, small RNA is able to move towards the younger shoot parts by following the mesophyll cell—bundle sheath parenchyma cell—phloem route (Kehr and Buhtz 2007). Based on the results of molecular examinations, it can be stated that the spread towards the younger shoot parts had already taken place at the time of sampling (Balassa et al. 2021, 2022). This is also indicated by the significantly higher RNAi gene expression measured in the youngest leaves of the *siRNA* group compared to the control: 13 of the 16 genes examined had a measurable effect at the molecular level one day after siRNA treatment (Fig. 3). This trend in the qPCR data of the *siRNA* group indicates that the RNAi system had been activated before the appearance of the infection, confirming the successful priming effect of the siRNA treatment. The fact that the expression of certain plant genes can be influenced by well-designed, sequence-specific exogenous dsRNA, miRNA or siRNA treatment has already been proved in several experimental settings. For example, it was possible to reduce the level of *EGFP* and *NRTII* transcripts in overproducing transgenic *Arabidopsis* by means of sequence-specific dsRNA treatment (Dubrovina et al. 2019). Betti et al. (2021) showed that 24 h after treatment with exogenous *miR399* and *miR156* molecules, the expression of the target *PHO2* and *SPL9* genes was also downregulated. In addition, they raised the possibility that secreted miRNAs might act as signalling molecules between neighbouring plants, triggering PTGS in the receiving plant, through the influence of *AGO1* and *RDR6*. Exogenous dsRNA can also act as a biopesticide: Biedenkopf et al. (2020) showed that exogenous dsRNA introduced into barley caused a 60% decrease in the *SHP* gene expression of the barley pest S*itobion avenae*. It was also claimed that understanding of the underlying mechanisms of RNA-induced plant protection methods may have a significant potential for agronomic use.

At the onset of infection, there is a characteristic increase in the general gene expression of the RNAi system. The *AGO*, *DCL* and *RDR* genes examined in the present work could be classified into three main trend groups based on their behaviour: continuously increasing, decreasing and V-shaped trends. Among these, the most populous group consisted of 8 genes (*AGO1c*, *AGO2a*, *AGO2b*, *AGO18a*, *DCL1*, *DCL3a*, *MOP1* and *RDR1*) with constantly increasing expression activity, which presumably play an important role in the development of protection against MDMV in the sweet corn variety tested. According to the heat map, *AGO18a*, *AGO4b*, *DCL4*, *RDR6* and *MOP1* form a well-separated cluster. Yang and Li (2018) summarised the possible antiviral mechanisms of RNAi. Among others they listed RDR6, DCL2/3/4 and several AGOs as key elements in antiviral defence. RDR6 is crucial in secondary siRNA biogenesis, while DCL2/3/4 generates viral siRNA, which then contributes to the slicing or translational inhibition of viral RNA. The *MOP1* gene is an *RDR2* homologue and participates in the establishment and maintenance of paramutations and transcriptional silencing (Alleman et al. 2006). The MOP1/DCL3 and RDR6/DCL4 siRNA pathways were reported to maintain the production of the 22 nt siRNAs that are required for AGO4-mediated RdDM (Nobuta et al. 2008). Both small RNA treatment and MDMV infection had a significant effect on gene expression, from which it can be concluded that they play a central role in antiviral defence against MDMV. It is important to emphasise that when MDMV infection is mitigated by small RNA, the activity of the RNAi genes shows a similar character to the *Inf* group, but typically has a significantly lower value. This can be explained by the fact that the RNAi system in the *siRNA-Inf* group was prepared for possible infection as a result of the priming effect, so presumably a much faster, more efficient and less energy-demanding defence took place compared to the *Inf* group. The best evidence for this is the expression activity of *AGO18a*, where the largest (16 × fold change) increase occurred as a result of infection, while it remained well below that in the *siRNA-Inf* group.

Pearson’s correlation analysis provides information about the relationship between the genes examined with the qPCR technique. In terms of gene expression, *RDR1*, *DCL1*, *AGO1c* and *AGO1e* showed a positive correlation with each other, while at the trend level they were characterised by slightly inhibited expression, indicating their downregulation compared to the other genes. In the case of *AGO18a*, no significant correlation was found with the other genes, though weak or moderate positive interactions could be detected. This may indicate that it is related to the entire system, but that no single gene alone has a significant effect on it. *AGO18a* is a monocot-specific gene, whose expression responds to viral infections and plays a major role in the antiviral defence of infected tissues (Wu et al. 2015). In the case of rice stripe virus infection, rice AGO18 releases AGO1 from inhibition by binding *miR168*, thereby stimulating its accumulation. In addition, by binding *miR528*, it increases the activity of L-ascorbate oxidase (AO), which results in the accumulation of reactive oxygen species and thus the strengthening of antiviral defence (Wang et al. 2021). The *DCL3a* gene can also be highlighted from the correlation matrix, as it has only positive, moderate or strong relationships with all the other genes, while its expression profile shows dual, early and late activation.

Due to the priming resulting from the small RNA treatment, strong RNAi gene expression changes were therefore detected, in concordance with the accumulation of viral particles. During the monitoring of MDMV genetic material and coat protein, the amount of MDMV RNA in the *siRNA-Inf* group was found to remain significantly lower compared to the *Inf* group. The maximum amount of coat protein was also significantly lower in the *siRNA-Inf* group and its increase was delayed by one week compared to the *Inf* group. Thus, in the initial stage of MDMV infection, large-scale protein synthesis slowed down due to the siRNA pre-treatment, which hindered the development of the reproduction rate necessary for appropriate infection. Another interesting observation was that at 14 dpi, similar quantities of coat protein were detected in both groups, mirroring the findings at 5 dpi. Although the virus content of the root was not examined, this resemblance may suggest a temporary oscillation of the virus between the plant stem and root. The results align well with earlier findings, demonstrating that MDMV-specific siRNA pre-treatment effectively reduced the viral load. Consequently, the sweet corn plants exhibited less stunted development (one additional leaf level compared to the *Inf* group) and improved long-term physiological status (Ludmerszki et al. 2017; Balassa et al. 2021). Numerous data reported in the literature support the decrease in viral load after exogenous RNA treatments. For example, spraying with a solution containing several chilli leaf curl virus (ChiLCV)-specific dsRNAs successfully reduced the amount of ChiLCV DNA, helping to reduce disease incidence in tobacco plants by more than 50% (Singh et al. 2022). Virus-specific dsRNA treatment was also effective against the most aggressive pathogen of ghost peppers, the cucumber mosaic virus, as proved by the significantly lower severity of the symptoms on treated plants (Routhu et al. 2022). Certain stress factors, such as heat stress, can trigger permanent changes via epigenetics, as a result of which the defence system can be activated more quickly in the event of repeated stress (Ohama et al. 2017). The epigenetic changes also play an important role in case of biotic stresses, such as viral infections. The RNA-dependent DNA Methylation pathway, which is responsible for the regulation of transposable elements, and histone modifications are also necessary for the development of appropriate antiviral defence. In their absence, increased susceptibility to viral infection appears (Corrêa et al. 2020). The potential for MDMV-specific small RNA treatment to cause inheritable epigenetic changes remains unexplored. However, further investigation into this area could significantly broaden our understanding and open up new perspectives in the field of research.

Overall, it can be stated that the MDMV CP gene*-*derived siRNA pre-treatment tested here proved to be effective against MDMV infection. Due to the entry of siRNA and its priming effect, faster, more powerful antiviral defence was achieved, making it possible to slow down the infection. Further possibilities to achieve a stronger antiviral response are, among others, the recovery of more effective siRNA sequences on multiple siRNA sequences, the use of siRNA molecules bound to special carriers or the development of spraying treatment in which the exogenous siRNA is only one of the effective agents. Further interesting results can be expected in the future, concerning the clarification of the mechanism by which exogenous RNA molecules enter the mesophyll symplast.

## Conclusion

An accurate understanding of the initial stage of plant virus infection could effectively contribute to successful plant defence against the virus, since successfully controlled viral reproduction in the early phase of the infection fundamentally affects the outcome of the disease. By strengthening the plant defence system, i.e. RNA interference, there is a greater chance of effective plant protection. Pre-infection siRNA treatment provides an excellent opportunity for this. The presence of the fluorophore-bound siRNA after treatment was confirmed in samples taken from the treatment sites, using a fluorescent laser scanning microscope to detect the specific signal of the ALEXA488 fluorophore linked to the siRNA. Once the MDMV-specific siRNA entered the plant cell, it was able to promote antiviral protection. Similar to other studies, the applied siRNA pre-treatment triggered the activation of antiviral RNAi genes, while also contributing to diminishing the viral genetic material. This led to a one-week delay in the peak of infection in the siRNA pre-treated group. Based on the results obtained, it can be concluded that the pre-infection treatment with exogenous MDMV coat protein-derived siRNA can effectively strengthen the antiviral stress response of the tested sweet corn hybrid, potentially leading to an improvement in the physiological status and yield.

### Supplementary Information

Below is the link to the electronic supplementary material.

Supplementary file1 (MP4 13461 KB)

Supplementary file2 (MP4 17191 KB)

Supplementary file3 (MP4 16485 KB)

Supplementary file4 (TIFF 9813 KB)

Supplementary file5 (TIF 43085 KB)

Supplementary file6 (TIF 49301 KB)

Supplementary file7 (TIF 50160 KB)

Supplementary file8 (TIF 13416 KB)

Supplementary file9 (TIFF 28156 KB)

Supplementary file10 (DOCX 7 KB)

Supplementary file11 (XLSX 9 KB)

Supplementary file12 (XLSX 7 KB)

Supplementary file13 (XLSX 6 KB)

Supplementary file14 (XLSX 15 KB)

## Data Availability

All data generated or analysed during this study are included in this published article [and its supplementary information files]. All software applied in this study is cited in this published article.

## References

[CR1] Alleman M, Sidorenko L, McGinnis K, Seshadri V, Dorweiler JE, White J, Sikkink K, Chandler VL (2006) An RNA-dependent RNA polymerase is required for paramutation in maize. Nat 442:295–298. 10.1038/nature04884 10.1038/nature04884 16855589

[CR2] Balassa K, Balassa G, Gondor OK, Janda T, Almási A, Rudnóy S (2021) Changes in physiology, gene expression and ethylene biosynthesis in MDMV-infected sweet corn primed by small RNA pre-treatment. Saudi J Biol Sci 28:5568–5578. 10.1016/j.sjbs.2021.05.073 34588867 10.1016/j.sjbs.2021.05.073 PMC8459037

[CR3] Balassa G, Balassa K, Janda T, Rudnóy S (2022) Expression pattern of RNA interference genes during drought stress and MDMV infection in maize. J Plant Growth Regul 41:2048–2058. 10.1007/s00344-022-10651-z 10.1007/s00344-022-10651-z

[CR4] Bennett M, Deikman J, Hendrix B, Iandolino A (2020) Barriers to efficient foliar uptake of dsRNA and molecular barriers to dsRNA activity in plant cells. Front Plant Sci 11:816. 10.3389/fpls.2020.00816 32595687 10.3389/fpls.2020.00816 PMC7304407

[CR5] Betti F, Ladera-Carmona MJ, Weits DA, Ferri G, Iacopino S, Novi G, Svezia B, Kunkowska AB, Santaniello A, Piaggesi A, Loreti E, Perata P (2021) Exogenous miRNAs induce post-transcriptional gene silencing in plants. Nat Plants 7:1379–1388. 10.1038/s41477-021-01005-w 34650259 10.1038/s41477-021-01005-w PMC8516643

[CR6] Biedenkopf D, Will T, Jelonek L, Furch ACU, Busche T, Koch A (2020) Systemic spreading of exogenous applied RNA biopesticides in the crop plant *Hordeum vulgare*. ExRNA 2:12. 10.1186/s41544-020-00052-3 10.1186/s41544-020-00052-3

[CR7] Brosnan CA, Voinnet O (2011) Cell-to-cell and long-distance siRNA movement in plants: mechanisms and biological implications. Cur Opinion in Plant Biol 14:580–587. 10.1016/j.pbi.2011.07.011 10.1016/j.pbi.2011.07.011 21862389

[CR8] Clark MF, Adams AN (1977) Characteristics of the microplate method of enzyme-linked immunosorbent assay for the detection of plant viruses. J Gen Virol 34:475–483. 10.1099/0022-1317-34-3-475 323416 10.1099/0022-1317-34-3-475

[CR9] Corrêa RL, Sanz-Carbonell A, Kogej Z, Müller SY, Ambrós S, López-Gomollón S, Gómez G, Baulcombe DC, Elena SF (2020) Viral fitness determines the magnitude of transcriptomic and epigenomic reprograming of defense responses in plants. Mol Biol and Evol 37(7):1866–1881. 10.1093/molbev/msaa091 32259238 10.1093/molbev/msaa091

[CR10] Dalakouras A, Wassenegger M, McMillan JN, Cardoza V, Maegele I, Dadami E, Runne M, Krczal G, Wassenegger M (2016) Induction of silencing in plants by high-pressure spraying of in vitro-synthesized small RNAs. Front Plant Sci 7:1327. 10.3389/fpls.2016.01327 27625678 10.3389/fpls.2016.01327 PMC5003833

[CR11] Dalakouras A, Wassenegger M, Dadami E, Ganopoulos I, Pappas ML, Papadopoulou K (2020) Genetically modified organism-free RNA interference: exogenous application of RNA molecules in plants. Plant Physiol 182:38–50. 10.1104/pp.19.00570 31285292 10.1104/pp.19.00570 PMC6945881

[CR12] Demirer GS, Zhang H, Matos JL, Goh NS, Cunningham FJ, Sung Y, Chang R, Aditham AJ, Chio L, Cho MJ, Staskawicz B, Landry MP (2019) High aspect ratio nanomaterials enable delivery of functional genetic material without DNA integration in mature plants. Nat Nanotechnol 14:456–464. 10.1038/s41565-019-0382-5 30804481 10.1038/s41565-019-0382-5 PMC10461892

[CR13] Demirer GS, Zhang H, Goh NS, Pinals RL, Chang R, Landry MP (2020) Carbon nanocarriers deliver siRNA to intact plant cells for efficient gene knockdown. Sci Adv 6:2375–2548. 10.1126/sciadv.aaz0495 10.1126/sciadv.aaz0495 PMC7314522 32637592

[CR14] Dubrovina AS, Aleynova OA, Kalachev AV, Suprun AR, Ogneva ZV, Kiselev KV (2019) Induction of transgene suppression in plants via external application of synthetic dsRNA. Int J Mol Sci 20:1585. 10.3390/ijms20071585 30934883 10.3390/ijms20071585 PMC6479969

[CR15] Dubrovina AS, Aleynova OA, Suprun AR, Ogneva ZV, Kiselev KV (2020) Transgene suppression in plants by foliar application of in vitro-synthesized small interfering RNAs. Appl Microbiol Biotechnol 104:2125–2135. 10.1007/s00253-020-10355-y 31932895 10.1007/s00253-020-10355-y

[CR16] Gao L, Luo J, Ding J, Wang T, Hu T, Song P, Zhai R, Zhang H, Zhang K, Li K, Zhi H (2019) Soybean RNA interference lines silenced for eIF4E show broad potyvirus resistance. Mol Plant Pathol 21:303–317. 10.1111/mpp.12897 31860775 10.1111/mpp.12897 PMC7036369

[CR17] Guo AY, Zhang YM, Wang L, Bai D, Xu YP, Wu WQ (2021) Single-molecule imaging in living plant cells: a methodological review. Int J Mol Sci 22:5071. 10.3390/ijms22105071 34064786 10.3390/ijms22105071 PMC8151321

[CR18] Hameed A, Tahir MN, Asad S, Bilal R, Eck JV, Jander G, Mansoor S (2017) RNAi-mediated simultaneous resistance against three RNA viruses in potato. Mol Biotechnol 59:73–83. 10.1007/s12033-017-9995-9 28194691 10.1007/s12033-017-9995-9

[CR19] Hipper C, Brault V, Ziegler-Graff V, Revers F (2013) Viral and cellular factors involved in phloem transport of plant viruses. Front Plant Sci 4:154. 10.3389/fpls.2013.00154 23745125 10.3389/fpls.2013.00154 PMC3662875

[CR20] Hong H, Wang C, Huang Y, Xu M, Yan J, Feng M, Li J, Shi Y, Zhu M, Shen D, Wu P, Kormelink R, Tao X (2021) Antiviral RISC mainly targets viral mRNA but not genomic RNA of tospovirus. PLoS Pathog 17:e1009757. 10.1371/journal.ppat.1009757 34320034 10.1371/journal.ppat.1009757 PMC8351926

[CR21] Jin Y, Zhao JH, Guo HS (2021) Recent advances in understanding plant antiviral RNAi and viral suppressors of RNAi. Cur Op in Virol 46:65–72. 10.1016/j.coviro.2020.12.001 10.1016/j.coviro.2020.12.001 33360834

[CR22] Kannan M, Ismail I, Bunawan H (2018) *Maize dwarf mosaic virus*: from genome to disease management. Viruses 10:492. 10.3390/v10090492 30217014 10.3390/v10090492 PMC6164272

[CR23] Kaldis A, Berbati M, Melita O, Reppa C, Holeva M, Otten P, Voloudakis A (2018) Exogenously applied dsRNA molecules deriving from the Zucchini yellow mosaic virus (ZYMV) genome move systemically and protect cucurbits against ZYMV. Mol Plant Pathol 19:883–895. 10.1111/mpp.12572 28621835 10.1111/mpp.12572 PMC6638139

[CR24] Kehr J, Buhtz A (2007) Long distance transport and movement of RNA through the phloem. J Exp Bot 59:85–92. 10.1093/jxb/erm176 17905731 10.1093/jxb/erm176

[CR25] Koch A, Biedenkopf D, Furch A, Weber L, Rossbach O, Abdellatef E, Linicus L, Johannsmeier J, Jelonek L, Goesmann A, Cardoza V, McMillan J, Mentzel T, Kogel KH (2016) An RNAi-based control of *Fusarium graminearum* infections through spraying of long dsRNAs involves a plant passage and is controlled by the Fungal Silencing Machinery. PLoS Pathog 12:e1005901. 10.1371/journal.ppat.1005901 27737019 10.1371/journal.ppat.1005901 PMC5063301

[CR26] Koressaar T, Remm M (2007) Enhancements and modifications of primer design program Primer3. Bioinformatics 23:1289–1291. 10.1093/bioinformatics/btm091 17379693 10.1093/bioinformatics/btm091

[CR27] Koukiekolo R, Jakubek ZJ, Cheng J, Sagan SM, Pezacki JP (2009) Studies of a viral suppressor of RNA silencing p19-CFP fusion protein: A FRET-based probe for sensing double-stranded fluorophore tagged small RNAs. Biophysical Chem 143:166–169. 10.1016/j.bpc.2009.05.001 10.1016/j.bpc.2009.05.001 19491057

[CR28] Ludmerszki E, Chounramany S, Oláh C, Kátay G, Rácz I, Almási A, Solti Á, Bélai I, Rudnóy S (2017) Protective role of S-methylmethionine-salicylate in maize plants infected with *Maize dwarf mosaic virus*. Eur J Plant Pathol 149:145–156. 10.1007/s10658-017-1174-0 10.1007/s10658-017-1174-0

[CR29] Mitter N, Worrall EA, Robinson KE, Robinson KE, Li O, Jain RG, Taochy C, Fletcher SJ, Carroll BJ, Lu GQ, Xu ZP (2017) Clay nanosheets for topical delivery of RNAi for sustained protection against plant viruses. Nat Plants 3:16207. 10.1038/nplants.2016.207 28067898 10.1038/nplants.2016.207

[CR30] Mohamed A, Jin Z, Osman T, Shi N, Tör M, Jackson S, Hong Y (2022) Hotspot siRNA confers plant resistance against viral infection. Biology 11:714. 10.3390/biology11050714 35625441 10.3390/biology11050714 PMC9138956

[CR31] Nobuta K, Lu C, Shrivastava R, Pillay M, Paoli ED, Accerbi M, Arteaga-Vazquez M, Sidorenko L, Jeong DH, Yen Y, Green PJ, Chandler VL, Meyers BC (2008) Distinct size distribution of endogenous siRNAs in maize: evidence from deep sequencing in the mop1-1 mutant. PNAS 105:14958–14963. 10.1073/pnas.0808066105 18815367 10.1073/pnas.0808066105 PMC2567475

[CR32] Ohama N, Sato H, Shinozaki K, Yamaguchi-Shinozaki K (2017) Transcriptional Regulatory Network of Plant Heat Stress Response. Trends in Plant Sci 22(1):53–65. 10.1016/j.tplants.2016.08.015 27666516 10.1016/j.tplants.2016.08.015

[CR33] Pfaffl MW, (2004) Quantification strategies in real time PCR, in: A-Z of Quantitative PCR. In: Bustin SA (ed) International University Line, CA, La Jolla, 87–112

[CR34] Qiao L, Lan C, Capriotti L, Ah-Fong A, Sanchez JN, Hamby R, Heller J, Zhao H, Glass NL, Judelson HS, Mezzetti B, Niu D, Jin H (2021) Spray-induced gene silencing for disease control is dependent on the efficiency of pathogen RNA uptake. Plant Biotechnol J 19:1756–1768. 10.1111/pbi.13589 33774895 10.1111/pbi.13589 PMC8428832

[CR35] Racine JS (2012) Rstudio: a platform-independent IDE for r and Sweave. J Applied Eco 27:167–172. 10.1002/jae.1278 10.1002/jae.1278

[CR36] Ramakers C, Ruijter JM, Deprez RHL, Moorman AFM (2003) Assumption-free analysis of quantitative real-time polymerase chain reaction (PCR) data. Neurosci Lett 339:62–66. 10.1016/S0304-3940(02)01423-4 12618301 10.1016/S0304-3940(02)01423-4

[CR37] Redinbaugh MG, Stewart LR (2018) Maize lethal necrosis: an emerging, synergistic viral disease. Annu Rev Virol 5:301–322. 10.1146/annurev-virology-092917-043413 30059641 10.1146/annurev-virology-092917-043413

[CR38] Rego-Machado CM, Nakasu EYT, Silva JMF, Lucinda N, Nagata T, Inoue-Nagata AK (2020) siRNA biogenesis and advances in topically applied dsRNA for controlling virus infections in tomato plants. Sci Rep 10:22277. 10.1038/s41598-020-79360-5 33335295 10.1038/s41598-020-79360-5 PMC7746768

[CR39] Routhu G, Borah M, Siddappa S, Nath PD (2022) Exogenous application of coat protein-specifc dsRNA inhibits cognate *cucumber mosaic virus* (CMV) of ghost pepper. J Plant Dis Prot 129:293–300. 10.1007/s41348-021-00558-4 10.1007/s41348-021-00558-4

[CR40] Schwartz SH, Hendrix B, Hoffer P, Sanders RA, Zheng W (2019) Carbon dots for efficient siRNA delivery and gene silencing in plants. Plant Physiol 184:647–657. 10.1104/pp.20.00733 10.1104/pp.20.00733 PMC7536711 32764133

[CR41] Singh OW, Gupta D, Joshi B, Roy A, Mukherjee SK, Mandal B (2022) Spray application of a cocktail of dsRNAs reduces infection of Chilli leaf curl virus in *Nicotiana benthamiana*. J Plant Dis Prot 129:433–438. 10.1007/s41348-021-00549-5 10.1007/s41348-021-00549-5

[CR42] Wang L, Xie H, Zheng X, Chen J, Zhang S, Wu J (2021) Recent advances and emerging trends in antiviral defense networking in rice. The Crop J 9:553–563. 10.1016/j.cj.2021.02.009 10.1016/j.cj.2021.02.009

[CR43] Wu J, Yang Z, Wang Y, Zeng L, Ye R, Ji Y, Zhao S, Ji S, Liu R, Xu L, Zheng H, Zhou Y, Zhang X, Cao X, Xie L, Wu Z (2015) Viral-inducible Argonaute18 confers broad-spectrum virus resistance in rice by sequestering a host microRNA. eLife 4: e05733. 10.7554/eLife.05733 10.7554/eLife.05733 PMC4358150 25688565

[CR44] Xia Z, Peng J, Li Y, Chen L, Li S, Zhou T, Fan Z (2014) Characterization of small interfering RNAs derived from sugarcane mosaic virus in infected maize plants by deep sequencing. PLoS ONE 9:e97013. 10.1371/journal.pone.0097013 24819114 10.1371/journal.pone.0097013 PMC4018358

[CR45] Yang Z, Li Y (2018) Dissection of RNAi-based antiviral immunity in plants. Cur Op in Virol 32:88–99. 10.1016/j.coviro.2018.08.003 10.1016/j.coviro.2018.08.003 30388659

