# Supplementary figures and images for: Detection of exogenous siRNA inside sweet corn bundle sheath cells and the RNAi dynamics in the early stage of Maize dwarf mosaic virus infection
Source: Physiol Mol Biol Plants. 2024 Aug 14;30(8):1265–76. doi: 10.1007/s12298-024-01500-2 (PMC11341793; doi:10.1007/s12298-024-01500-2)

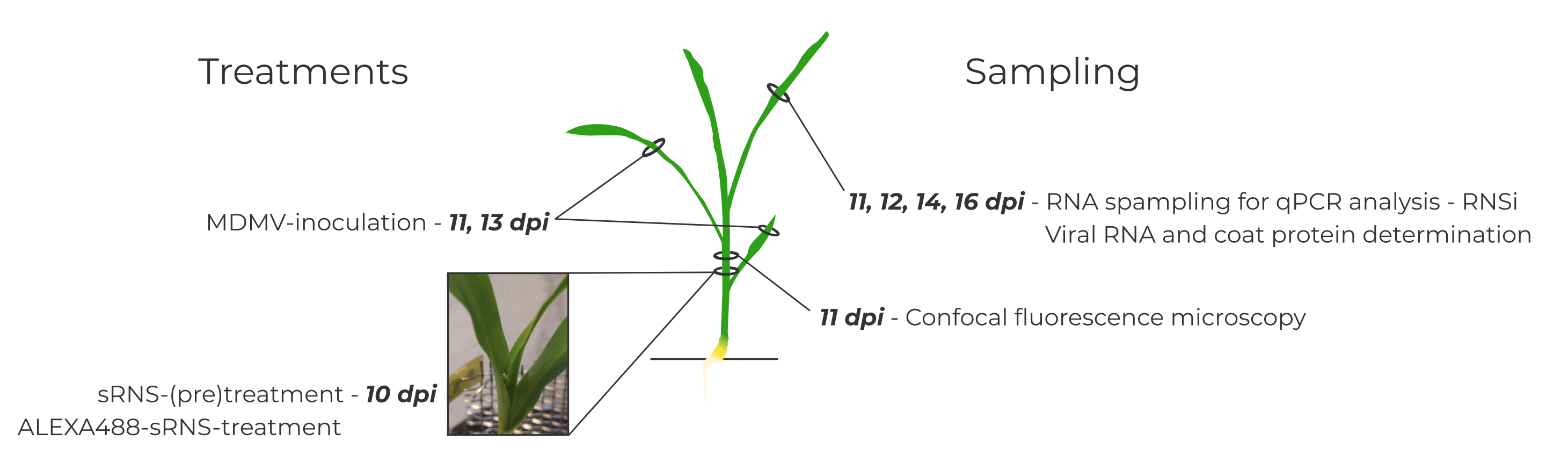

Supplement: Supplementary file 4 — Supplementary file4 (TIFF 9813 KB) [file 12298_2024_1500_MOESM4_ESM.tiff]

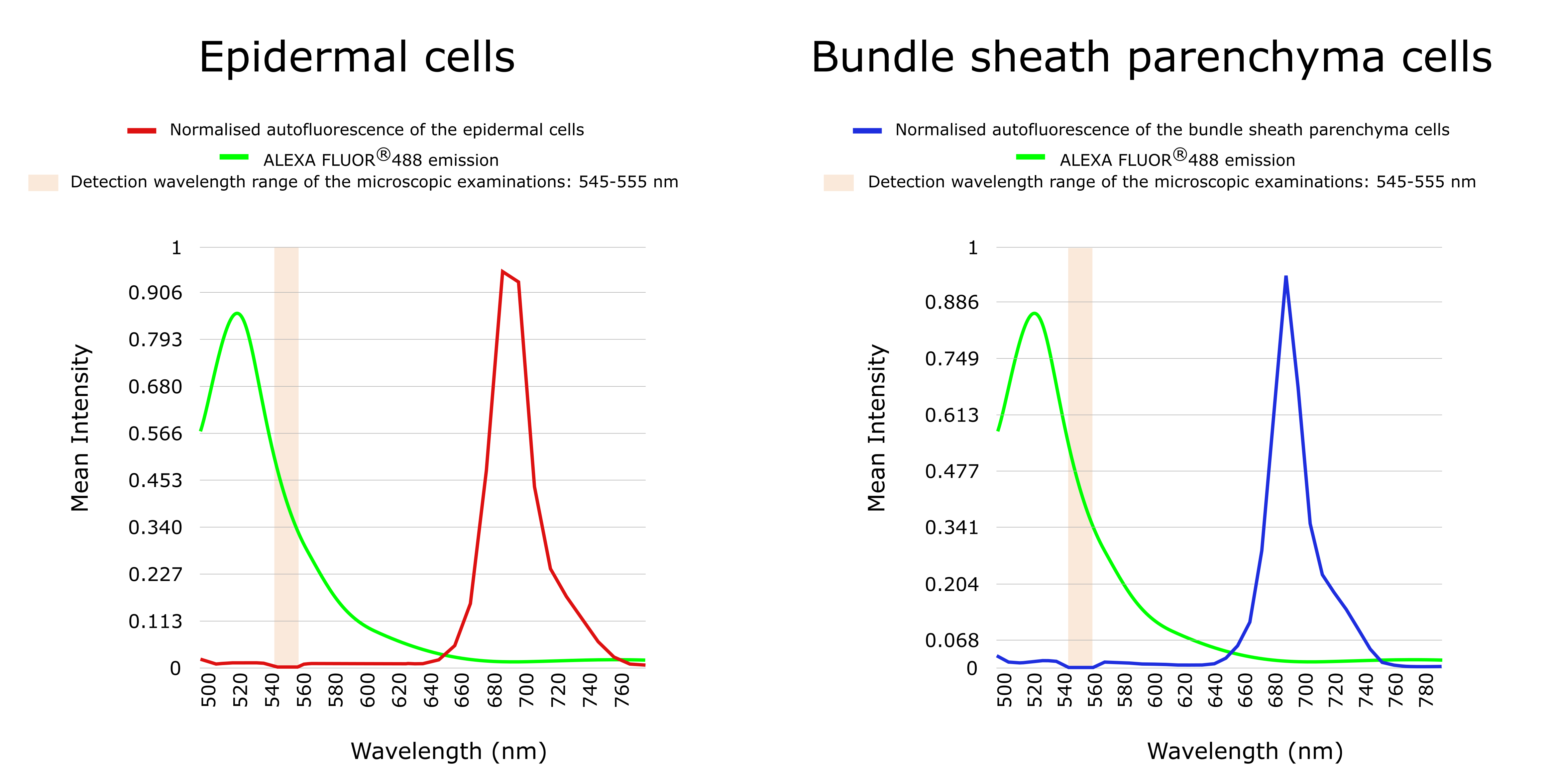

Supplement: Supplementary file 5 — Supplementary file5 (TIF 43085 KB) [file 12298_2024_1500_MOESM5_ESM.tif]

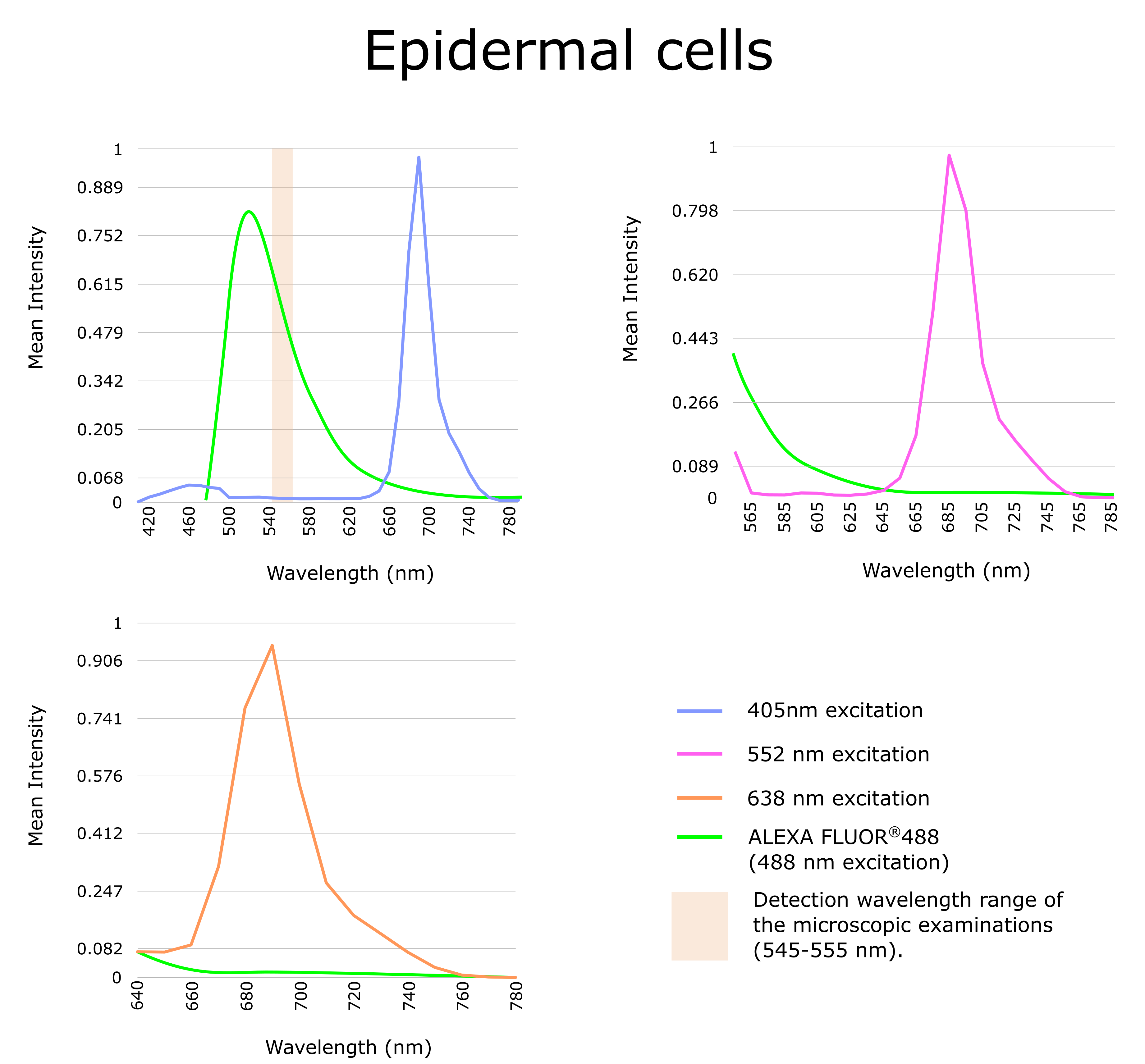

Supplement: Supplementary file 6 — Supplementary file6 (TIF 49301 KB) [file 12298_2024_1500_MOESM6_ESM.tif]

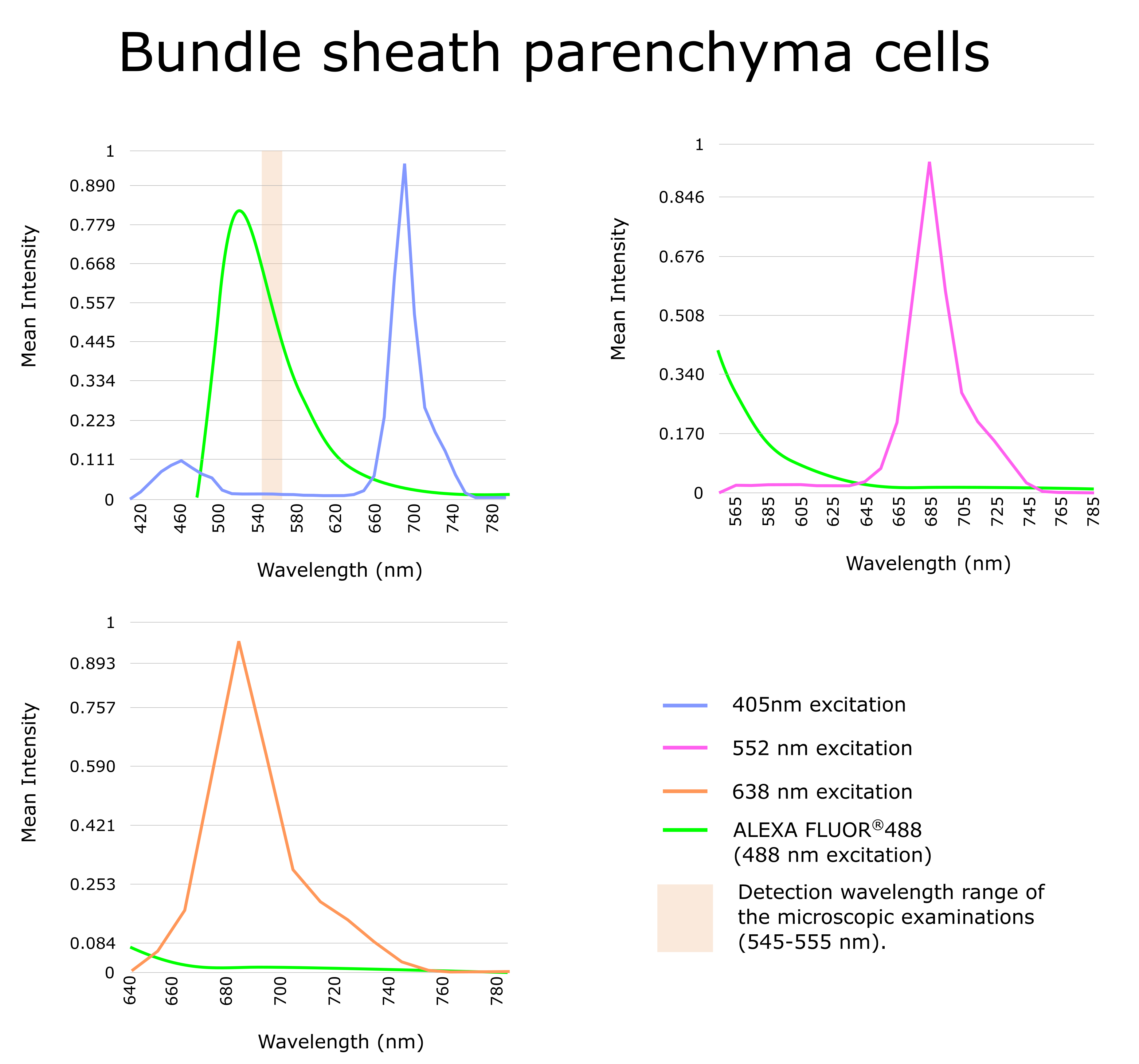

Supplement: Supplementary file 7 — Supplementary file7 (TIF 50160 KB) [file 12298_2024_1500_MOESM7_ESM.tif]

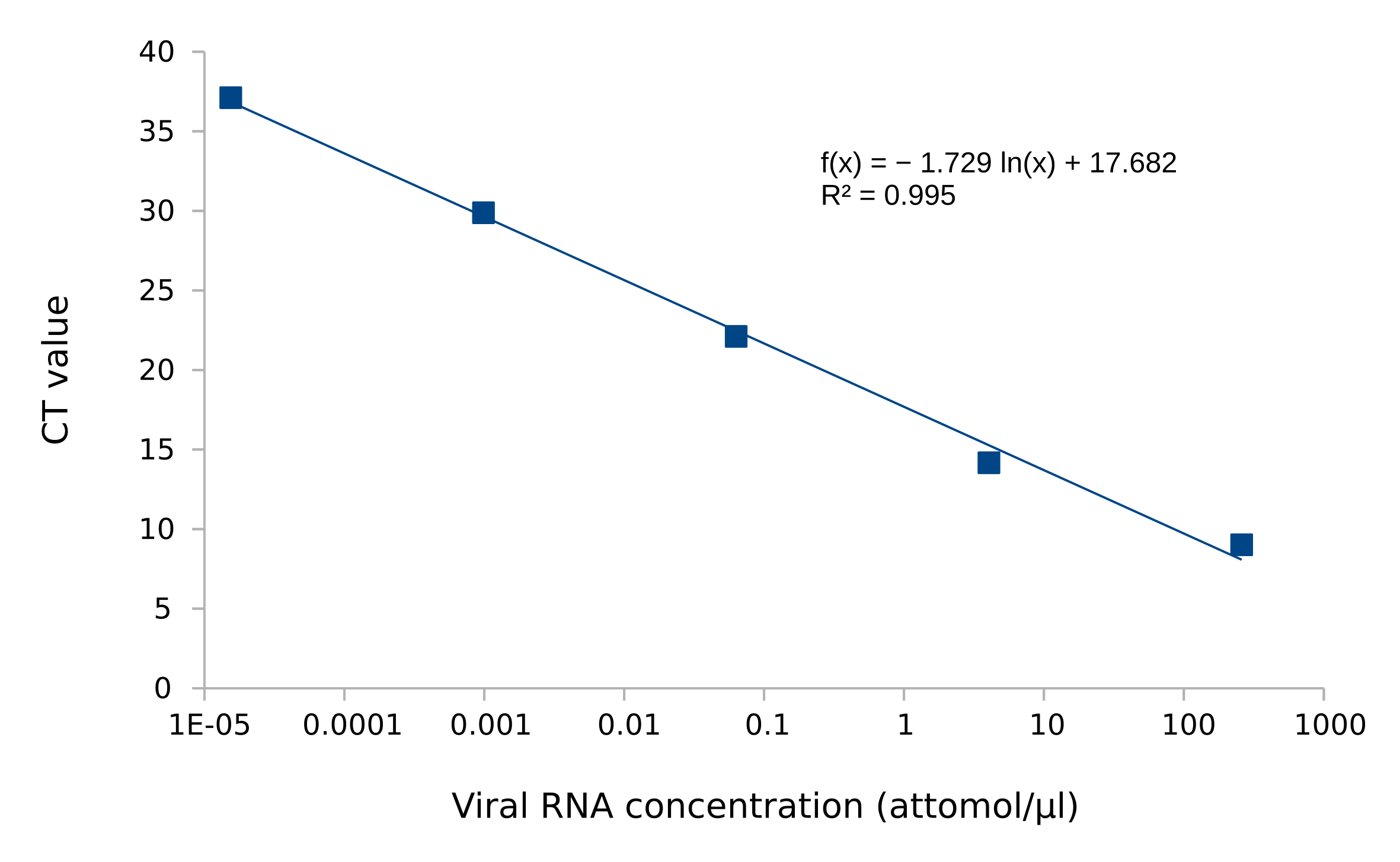

Supplement: Supplementary file 8 — Supplementary file8 (TIF 13416 KB) [file 12298_2024_1500_MOESM8_ESM.tif]

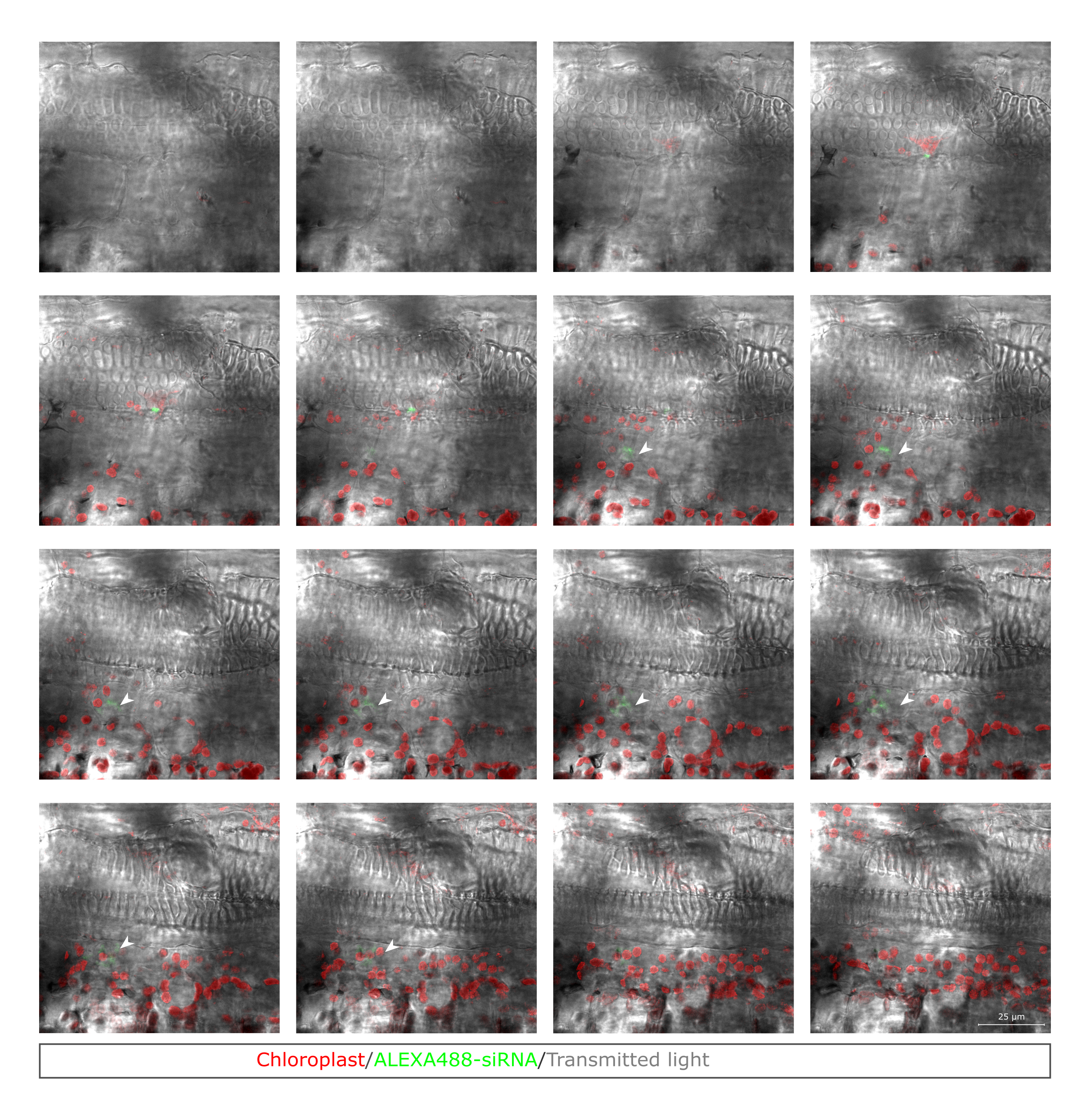

Supplement: Supplementary file 9 — Supplementary file9 (TIFF 28156 KB) [file 12298_2024_1500_MOESM9_ESM.tiff]
