# Supplementary material for: Detection of exogenous siRNA inside sweet corn bundle sheath cells and the RNAi dynamics in the early stage of Maize dwarf mosaic virus infection
Source: Physiol Mol Biol Plants. 2024 Aug 14;30(8):1265–76. doi: 10.1007/s12298-024-01500-2 (PMC11341793; doi:10.1007/s12298-024-01500-2)
Supplement: Supplementary file 10 — Supplementary file10 (DOCX 7 KB) [file 12298_2024_1500_MOESM10_ESM.docx]

**Supplementary Figure 1 -** The experimental set-up and sampling points of the tests performed (dpi - days post first infection).

**Supplementary Figure 2** - ALEXA FLUOR^®^488 emission and the normalized autofluorescence of targeted corn tissues under 488 nm excitation. The detection range (545-555 nm) used during the microscopic measurements is also shown

**Supplementary Figure 3 - Fig10** Normalized autofluorescence of targeted sweet corn epidermis tissues under 405, 552 and 638 nm excitation. The detection range (545-555 nm) used during the microscopic measurements and the ALEXA FLUOR^®^488 emission under 488 excitation are also shown

**Supplementary Figure 4 -** Normalized autofluorescence of the targeted sweet corn bundle sheath parenchyma tissues under 405, 552 and 638 nm excitation. The detection range (545-555 nm) used during the microscopic measurements and the ALEXA FLUOR^®^488 emission under 488 excitation are also shown

**Supplementary Figure 5 -**  Calibration line used to determine the absolute results of the PrimeTime tests.

**Supplementary Figure 6 -** Multi-panel figure of ALEXA488-siRNA treated plants, 3 mm from the treatment site (open leaf sheaths) 20 hours after treatment. Channels: red – chloroplast autofluorescence, gray – transmitted light, green – ALEXA488-siRNA (red excitation 638 nm, red detection 645-720 nm; green excitation 488 nm, green detection 535-565 nm)

**Online Resource 1 – ESM1** ALEXA FLUOR 488^®^ signal detected within intact cells in transversal sections taken 3 mm from the treatment site (open leaf sheaths) 20 hours after treatment. The red signal denotes the fluorescence of the chloroplasts in the bundle sheath parenchyma.

**Online Resource 2 – ESM2** 3D videos of the section field visible in the Video SM2a recording. Channels: red – chloroplast autofluorescence, cyan – cell wall autofluorescence.

**Online Resource 3 – ESM3** Representative recording of negative control samples that consistently lacked the ALEXA FLUOR 488® fluorescent signal. Channels: red – chloroplast autofluorescence, cyan – cell wall autofluorescence.
